# A review on EMG/EEG based control scheme of upper limb rehabilitation robots for stroke patients

**DOI:** 10.1016/j.heliyon.2023.e18308

**Published:** 2023-07-20

**Authors:** Saad M. Sarhan, Mohammed Z. Al-Faiz, Ayad M. Takhakh

**Affiliations:** a Department of Biomedical Engineering, College of Engineering, Al-Nahrain University, Baghdad, Iraq; b Department of Control and Computer, College of Information Engineering, Al-Nahrain University, Baghdad, Iraq; c Department of Biomechanics, College of Engineering, Al-Nahrain University, Baghdad, Iraq

**Keywords:** Stroke rehabilitation, EEG, EMG, Robotics, Upper limb exoskeleton

## Abstract

Stroke is a common worldwide health problem and a crucial contributor to gained disability. The abilities of people, who are subjected to stroke, to live independently are significantly affected since affected upper limbs' functions are essential for our daily life. This review article focuses on emerging trends in BCI-controlled rehabilitation techniques based on EMG, EEG, or EGM + EEG signals in the last few years. Working on developing rehabilitation robotics, is considered a wealthy scientific area for researchers in the last period. There is a significant advantage that the human acquires from the interaction between the machine and his body, rehabilitation for a patient's limb is very important to get the body limb recovery, and this is what is provided mostly by applying robotic devices.

## 1 Introduction

When the oxygenated blood supply to the brain is cut off, a potentially fatal condition known as a stroke results, and the linked brain cells begin to die after a short period of time [1]. Each year, around 12.2 million new strokes occur worldwide, with one stroke occurring every 3 s [2]. 80% of survivors have got physical disabilities according to the studies managed by stroke rehabilitation [3]. Stroke often leads to hemiplegia, in this case, paralysis happens to one side of the body, when just one cerebral hemisphere is affected. Catastrophic difficulties associated with Activities of Daily Life (ADL) are seen in stroke patients, which affect the independence and quality of the patient's life [[4], [5], [6]]. Stroke rehabilitation is a program of various therapies prepared to help the patient relearn expertise lost after a stroke. Rehabilitation can help with speech, movement, strength, and daily living tasks. With a designed rehabilitation program, the patient can regain independence and enhance his quality of life [7]. Normally, the rehabilitation plan will involve physical activities, cognitive and emotional activities, and technology-assisted physical activities [3]. Rehabilitation needs a qualified therapist to practice repeatedly using a limb that is compromised [8]. However, at the time of the therapeutic session, patients and therapists are impacted by the availability of the therapists and the price of the rehabilitation equipment [[9], [10]]. Moreover, Individual contacts between the patient and the therapist are necessary for rehabilitation programs. However, interactive therapy is time- and labor-intensive for the patient as well as the therapist [8]. These rehabilitation robots deliver repeat movements for a patient's limb while also providing accurate, thorough, quantitative, and safe therapy [11]. The development of a Brain-Controlled Interface (BCI) for rehabilitation uses a variety of technologies [12] such as: Electroencephalography (EEG), Functional Magnetic Resonance Imaging (fMRI) [13], Electromyography (EMG) [14], Targeted Muscle Reinnervation (TMR) [15] and Force Myography (FMG) [16]. Even though invasive intracortical recordings offer brain signals that are more accurate and have superior spatial resolution [17]. Researchers are focusing on non-invasive recording techniques like EEG because of the inherent surgical risks in invasive techniques [18]. Studies shown that the effectiveness of robotic devices is impacted by the feature extraction techniques [19]. The EMG signals are among the greatest bioelectrical signals that can be recorded on the skin surface and are particularly well suited for controlling robotic equipment [20]. Every muscle movement has a unique pattern of distinct muscle fibers being activated, hence multi-channel EMG recordings can be utilized to categorize the movement [21].

In this review, upper limb rehabilitation after stroke by using EMG, EEG, and EEG + EMG non-invasive BCI techniques will be presented. In the next section, a brief description of EEG and EMG signals. The types of rehabilitation treatments will be listed. Later, EMG and/or EEG-Based robotic exoskeleton, and EMG/EEG-based control system for upper limb rehabilitation techniques will be included.

## 2 Technologies for EEG/EMG activity monitoring

### 2.1 Electroencephalography (EEG) signal

EEG is a process for capturing the electrical activity that the brain generates on the scalp in order to explain the macroscopic activity of the brain's surface layer underneath. The electrodes are placed on the scalp, making it a non-invasive procedure [22]. Various other methods are existed to survey brain function, including Positron Emission Tomography (PET), (fMRI), Electrocorticography (ECoG), Magnetoencephalography (MEG), Nuclear Magnetic Resonance Spectroscopy (NMR or MRS), Near-Infrared Spectroscopy (NIRS), Single-Photon Emission Computed Tomography (SPECT), and an Event-Related Optical Signal (EROS). The one-dimensional signals from constrained peripheral head regions make EEG notable for its simple fidelity despite the relatively low spatial sensitivity that it is concerned with, and it has received significant basic and clinical research throughput [23]. Compared to some of the other techniques, EEG has some advantages [[24], [25], [26], [27], [28], [29], [30], [31], [32], [33], [34]]:

• It has lower hardware costs.
• Providing immediate patient care in high-traffic hospitals by the limited number of technologists is prevented through EEG.
• It only needs a calm place and a small amount of equipment, whereas other techniques require bulky and stationary equipment.
• It has a high temporal resolution.
• It tolerates subject movement comparatively less than other neuroimaging methods.
• It does not provoke claustrophobia.
• Exposure to very strong magnetic fields is excluded.
• Unlike positron emission tomography, There is no radioligand exposure in EEG.
• Processing concerned with EEG doesn't need a response.
• It can be applied to subjects who are incapable of responding motorically.
• In comparison to other research methods such as BOLD response in MRI, a better understanding of the signal being measured.

Despite many advantages of EEG techniques, there are some limitations like [22,26,35]:

• It has a poor spatial resolution.
• It doesn't capture axonal action potential directly.
• For a given EEG signal, it is mathematically impossible to reproduce a unique intracranial current origin.

The frequency range of the EEG rhythms determines how they are categorized as shown in Table I [36].

**Table 1:** Categorization of Eeg Rhythms Depending on their Frequencies.

Rhythm	Frequency range (Hz)	Amplitude (μV)	Brain region	Subject state
**Delta**	0.1–3.5	50–350	Central	Deep sleep
**Theta**	4–7.5	10–150	Central	Drowsiness and the first step of sleep
**Alpha**	8–13	20–100	posterior	Focusing with eyes closed
**Beta**	above 13	10–30	Central	Cognitive efforts, drowsiness, and light sleep
**Mu**	7–12	10–50	Central	Movement
**Lambda**	200–300	below 50	Occipital	Visual exploration

**Fig. 1:**
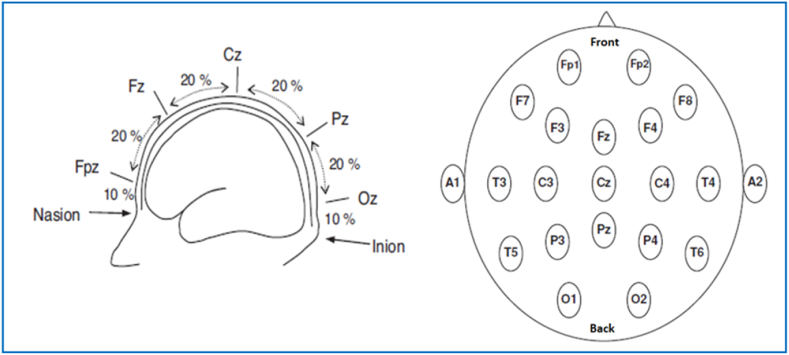
The international standard 10–20 system of EEG electrodes [36].

The placement of the electrode follows the international standard 10–20 system which stands for the distance percentage between neighboring electrodes being 10% or 20% of the skull's overall right-left or front-back distance, respectively as shown in Fig. 1.

**Fig. 2:**
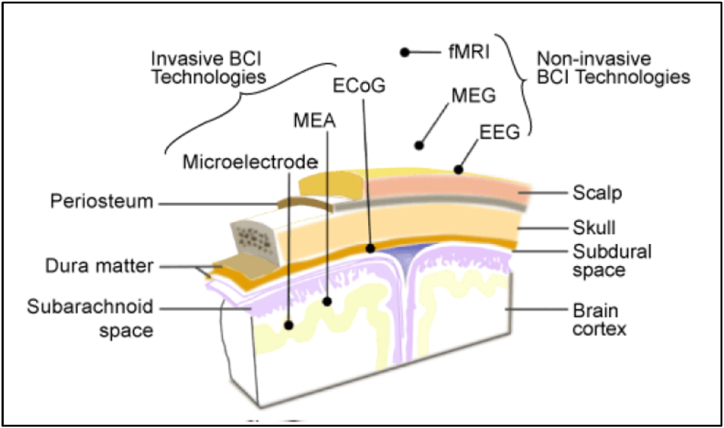
Electrodes position for various sensing technologies [37].

The typical electrode placement for different technologies of brain sensing is shown in Fig. 2.

### 2.2 Electromyography (EMG) signal

**Fig. 3:**
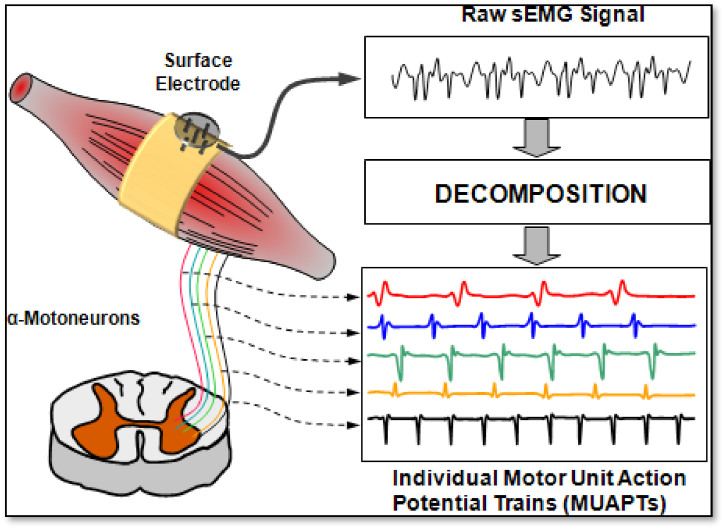
Physiological and anatomical muscle properties [38].

EMG is a tool used to perform EMG, and it creates a file called an electromyogram, which means that EMG, is an electrodiagnostic technique in medical applications to evaluate and record electrical activity produced by skeletal muscles [38]. This signal, which represents neuromuscular activity, is a biological signal that detects electrical currents generated during muscle contractions. The neurological system regulates the relaxation and contraction of muscles. Since the neurological system regulates the EMG signal and it depends on the physiological and anatomical characteristics of the muscle, it is a complex signal as shown in Fig. 3 [39]**.**

Analysis of EMG signals is crucial for biological applications and clinical diagnostics. Defining the realm of management and rehabilitation of motor disability is one of the key application areas [40]. Utilizing EMG signals in rehabilitation has some disadvantages such as insufficient remaining muscle activity and the existence of muscle fatigue [41]. On the contrary, Because EEG signals have a direct connection to the nervous system and can assess the activity of the brain during therapy, they have proven to be the most effective [42]. However, the Brain-Computer Interface (BCI) has some restrictions like accuracy and reliability when it is concerned with training for a complicated functional task [43]. A feasible solution to these difficulties is the combination of the benefits of both types of signals [44].

### 2.3 Assistive robotics or exoskeleton

**Fig. 4:**
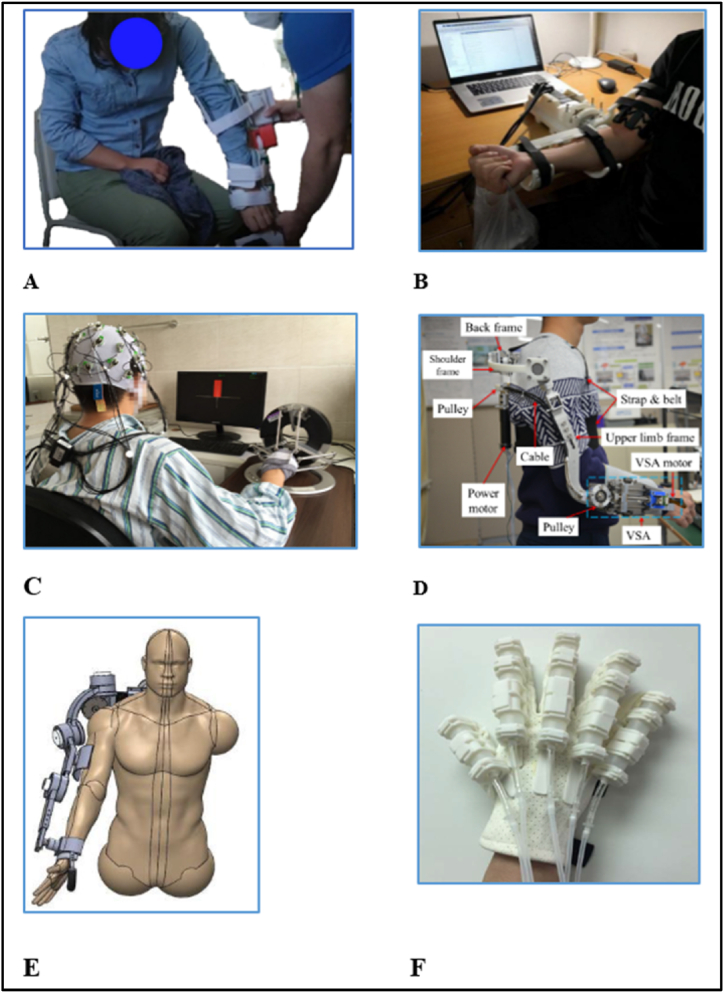
Various upper limb exoskeleton types (A) wrist and elbow exoskeleton [45], (B) arm exoskeleton rehabilitation robot [45], (C) Omega exoskeleton [46], (D) elbow exoskeleton [47], (E) CLEVERarm [48], (F) soft robotic fingers [49].

Robotic exoskeletons can be divided into three types according to their purpose, human efficiency enhancement exoskeletons, assistive devices, and therapeutic exoskeleton which is used for rehabilitation purposes and will be studied in this review. The robotic exoskeleton can be split into three main kinds depending on the body part involved: lower limb, upper limb, and particular joint exoskeleton. Fig. 4 illustrates many types of exoskeletons for upper limb rehabilitation.

## 3 Rehabilitation treatments

Selecting the best treatment approach is a crucial choice that will ultimately impact how well the treatment works [50]. Robots can give repetitive tasks to handicapped limbs [51]. There are three different categories of robot therapy [52].

### 3.1 Passive therapy

It is typically applied in the early phases of post-stroke symptoms, especially when the affected limb is not responding, and doesn't require any effort from the individual [53]. Patients with hemiplegia are typically advised to receive passive treatment [54]. It entails having a rehabilitation robot move the injured limb a certain number of times in a certain direction throughout the session [55].

### 3.2 Active therapy

It is used for patients who can move their affected limbs to some extent [54]. It is subdivided into two types: active-assistive therapy and active-resistive therapy. In the first one, the robot or therapist applies an external push to the joint to increase the range of motion while assisting the patient in doing the desired action [55]. Whereas the second type requires exerting opposing force on the damaged limbs via a robot or therapist [56].

### 3.3 Bilateral therapy

In rehabilitation, the mirroring principle is known as bilateral therapy [57]. A condition where the damaged limb moves similarly to an intact limb [58,59]. Bilateral therapy is provided via the Mirror Image Movement Enabler (MIME) and a few other robotic exoskeletons [60].

## 4 EMG-based control system for upper limb exoskeleton

There are different trends in EMG-Based robotic exoskeletons like increasing degree of freedom, decreasing weight, using soft material, and using different types of EMG sensors. In this section, current trends in the myoelectric activity control studies of the robotic exoskeletons such as elbow and wrist [45], arm [46], elbow joint [47,48], hand [61,62,63,64], elbow, wrist, and fingers [65], and wrist and hand [66] exoskeletons will be included.

An exoskeleton for the upper limbs that enables both mental and physical user interaction for active and passive sessions which promote the activation of neuroplasticity in the healing process for individuals who experience a neurological injury was presented. EMG signal features and classification models were assessed to determine the optimal settings for the cognitive human-robot interaction [45]. A lightweight upper arm exoskeleton robotic for rehabilitation based on EMG signals for home progressive resistive training was used. The overall exoskeleton's weight is about 3.03 kg only. The torque limit mode was employed to guarantee constant torque output at changing velocity. Principal component analysis was applied to improve the recognition accuracy to control the exoskeleton and provide muscle strength compensation while using k-NN to categorize (sEMG) signals under incremental training loads. This control method's dynamic recognition is 80.21% accurate [46]. sEMG-based robot-assisted bilateral training which achieved real-time stiffness modification depending on the dynamic motion of the subject [47]. Active exoskeleton for upper limb rehabilitation is employed to provide patients with very limited movement abilities with physical help to the required motion. The design provides one Degree of Freedom (DOF) active exoskeleton [48]. A multimodal sensing and interaction technique for hand rehabilitation was used to control hand movements. This sensory data is utilized as an input to the robotic hand orthosis to perform multiple tasks [61]. Soft hand exoskeletons actuated with Shape-Memory Alloys (SMA) wires were used as controlled robotic systems based on forearm muscle activity. Two movements were involved for patient rehabilitation purposes: grasping and releasing the objects [62]. Unique EMG-driven exoneuromusculoskeleton which combines soft pneumatic muscle and Neuromuscular Electrical Stimulation (NMES), for stroke survivors to practice using their upper limbs. Withdrawing and sequential arm reaching tasks can be achieved by the elbow, wrist, and fingers through the designed system depending upon EMG under voluntary effort control. The limb performance was enhanced when NMES and mechanical torque were provided. Significant improvements were recognized in the release of muscle spasticity and voluntary movement of the fingers, wrist, and elbow. The upper limb muscular coordination significantly improved after training. The findings suggested that the devised system can effectively facilitate stroke survivors' self-help upper limb rehabilitation [65]. A custom-made 2-channel low-cost EMG acquisition system was used to collect EMG signals, and placed on forearm muscles, and these signals were used to control a robotic hand. Open and close hand gestures were used. The integration of real-time, simply understood EMG-based visual feedback improves the subjects' performance. This feedback enables people to monitor their muscle activation in real time and, as a result, alter the force exerted. In contrast, due to lag times, kinesthetic feedback does not boost performance and cancels out the favorable influence of EMG-based visual feedback when both are present [63]. A cheap and 2-DOF portable robotic exoskeleton for stroke patients for wrist rehabilitation was implemented with a 3D printer technique utilizing Solid Works software program and Polylactic Acid (PLA) material and controlled with gyroscope and EMG MyoWare sensors. A considerable precise range of motion and velocity is presented [67]. The NeuroLife EMG System was developed and utilized, which comprises of a wearable forearm sleeve with 150 embedded electrodes and related software and hardware to record and decode sEMG. With an overall accuracy of 77.15.6%, 12 functional hand, wrist, and forearm movements were decoded, including various grasps from patients with various levels of chronic stroke damage [66]. Hand exoskeleton for essential functions in significant bilateral treatment was designed for subjects who have an injured right hand by cerebrovascular problems or an undesirable event that need passive or assisted rehabilitation. The subject's EMG signals were captured by a Myo Armband were received, processed, and classified through a Matlab GUI and achieved an accuracy of 81.2% utilizing the Random Subset Feature Selection (RSFS) algorithm for feature extraction and k-NN algorithm for classification. The patient's opposite hand's action is then reproduced by the exoskeleton. It features 8-DOF, was created using 3D printing, and the fingers may move freely. Fuzzy logic was used to control the movement [64]. A brief description of each rehabilitation robot in Table II.

**Table II:** A Brief Description of Emg-Based Upper Limb Exoskeleton.

Method Reference No; Year	Targeted Regions	No. of DOF	Types of Sensors Used	Advantages
Ref. [45] 2022	Elbow and wrist	3- DOF	9- sEMG electrodes and one load cell	Range of motion and user-exoskeleton coupling
Ref. [46] 2021	Arm	1- DOF	Myo Armband	Lightweight and home-based used
Ref. [47] 2019	Elbow joint	1- DOF	2- electrodes (Personal-EMG, Osaka Electronic Equipment Ltd., JAPAN)	Wearable and comfortable
Ref. [48] 2019	Elbow joint	1- DOF	MyoWare	The comfortable, lightweight, and sleek profile and strong material used
Ref. [61] 2018	Hand	1- DOF	Myo Armband	Effective force transmission and tendon-driven systems require less space
Ref. [62] 2018	Hand	1- DOF	Myo Armband	Light-weight and low-cost
Ref. [65] 2020	Elbow, wrist, and fingers	3- DOF	Pair of sEMG electrodes	Compact, lightweight and low-power requirement
Ref. [63] 2020	Hand	1- DOF	Pair of sEMG electrodes	Low cost and lightweight
Ref. [67] 2020	Wrist	2- DOF	MyoWare and gyroscope	Comfortable, lightweight, simple, and low cost
Ref. [66] 2020	Wrist and hand	6- DOF	150 embedded electrodes	Portable, comfortable and lightweight.
Ref. [64] 2022	Hand	8- DOF	Myo Armband	Low cost and allows for autonomous finger movement.

## 5 EEG-based control system for upper limb exoskeleton

In this section, studies involving robotic exoskeletons like hand [68,49,[69], [70], [71]], elbow, forearm, and wrist [72], and hand and fingers [73] exoskeletons that are controlled by EEG signals will be presented. Also, different sensors for brain activity and evaluation methods will be reviewed.

The clinical and subclinical effectiveness of the BCI was explored based on EEG signals and upper limb exoskeleton for subacute stroke therapy. 14 stroke subjects in the stage of subacute were participated and anyhow assigned to the control group (n = 7) and BCI group (n = 7). The BCI group underwent BCI treatment with exoskeleton response three times per week for four weeks. The Fugl–Meyer Assessment of Upper Extremity (FMA-UE) scale was utilized to evaluate the enhancement of motor function. Both the control group's (p = 0.048) and the BCI group's (p = 0.032) FMA-UE scores increased one month after the BCI intervention. Fewer patients in the control group (28.6%) experienced good motor recovery than in the BCI group (57.1%), and the control group (7.14%) demonstrated a lower percentage of improvement than the BCI group (12.77%) [68]. The soft robotic glove used in stroke hand rehabilitation was activated using user intention recognition based on Steady-State Visually Evoked Potentials (SSVEP). Thirty patients with stroke were enlisted, and they were split into three groups to receive robotic, BCI-robotic, and conventional therapy. Three stages of clinical evaluations using the Modified Ashworth Scale (MAS), the WMFT, and the FMA-UL were used: pre-training, post-training, and three months of follow-up. The BCI-robotic group exhibited important enhancement after training in FMA of shoulder and elbow (6.2 ± 5.94, p = 0.0004), FMA of wrist and hand (4.3 ± 2.83, p = 0.007), FMA full score (10.05 ± 8.03, p = 0.001), and WMFT (5.1 ± 5.53, p = 0.037) compared to other groups. Hand function recovery of SSVEP-BCI rehabilitation using a soft robotic glove expressed a superior outcome than a robotic glove alone [49]. Elbow exoskeleton (MAHI Exo-II) based on EEG was used. 10 chronic stroke patients participated in 12 treatment sessions including an elbow training BMI exoskeleton. Generally, each participant underwent 132 ± 22 repetitions in one session. With a 23 ± 20% rate of false positives, BMI accuracy was 79 ± 18% across all sessions and patients. Post-training clinical evaluations found that Action Research Arm Test (ARAT) scores and FMA-UE enhanced over baseline by5.35 ± 4.62 and 3.92 ± 3.73 respectively [72]. A low-cost 3D-printed wrist exoskeleton was designed and 11 healthy subjects participated. In comparison to the assessment prior to the BCI treatment, the Motor-Evoked Potentials (MEPs) raised by 35 ± 60% instantly after and 67 ± 60% 30 min after the BCI treatment. Results show that it is feasible to detect imagined movements with an open-source BCI setup and manage a cheap 3D-printed exoskeleton that, when utilized in conjunction with the BCI, can cause neural plasticity [74]. The Hand Exoskeleton for Rehabilitation Objectives (HERO) was designed to regain flexion and extension of fingers' tasks which are controlled by EEG. A lightweight, wearable exoskeleton was created using a three-dimensional (3D) printing technique and materials. This exoskeleton weighs only 102 g, making it comparable to everyday wear [69]. The feasibility of a treatment with upper limb robot-assisted hand orthosis for stroke recovery based on EEG was assessed, and the results were compared with the conventional therapy. Acute upper limb disability was present in 7 subacute and 3 chronic stroke subjects (M = 59.9 12.8) who were randomly assigned to undergo 1 month of BCI treatment and 1 month of traditional treatment. The results were composed of: The Action Research Arm Test (ARAT), FMA-UE, hand dynamometry, EEG, and transcranial magnetic stimulation (TMS)-derived motor evoked potentials. Measurements were conducted prior to and following each intervention. The ARAT and FMA-UE beyond BCI (8.4 ± 10; 23.1 ± 16) and after conventional therapy (8.7 ± 11; 21.9 ± 15) were greater (p < 0.017) in comparison to baseline (4.3 ± 6; 17.5 ± 15). The BCI's outstanding usability and minimal mental exertion were both reported by patients [70]. 20 sessions were performed by 18 hospitalized chronic stroke subjects and subsequently after a month. Five points of practical assessments were utilized: pre-1, pre-2, mid-3, post-4, and 1-month follow-up. They used the Wolf Motor Function Test (WMFT) as the primary end measure, while FMA scores for the wrist, hand, shoulder, and elbow were considered supplementary outcome measures. Neuroplasticity changes were evaluated using functional near-infrared spectroscopy (fNIRS) before and after 20 sessions of BCI therapy. Cross-time Functional Connectivity (FC) was correlated by Pearson correlation analysis. According to the results, there was a better functional outcome after BCI treatment and a 1-month follow-up. This included a higher likelihood of achieving a clinically significant increase in the WMFT full score compared to baseline, an increase in the FC between the ipsilateral and contralateral M1 following BCI treatment (P 0.05), and an increase in the FC between the contralateral M1 and the contralateral frontal lobe [71]. A new approach to intervention utilizing EEG activities was proposed to keep focusing on training by improving the participation of stroke victims utilizing robot-assisted treatment. This way is implemented by using an AMADEO rehabilitation device and applying it to stroke patients to perform 12 treatment sessions focused on hand-motor development. This gadget offers four different types of training programs with increased intensity, including passive treatment with biofeedback, passive treatment, active treatment, and active 2D treatment games. Eight electrodes were used to measure EEG signals to get Movement-Related Cortical Potential (MRCP) signals. The negative magnitude of the MRCP signals determines the functional hand recovery parameters. It has been demonstrated that each training mode demonstrates a different level of hand recovery, with the maximum recovery occurring when MRCP signals indicate that the patient is actively participating in the training [73]. A brief description of each exoskeleton is provided in Table III.

**Table III:** A Summarized Description of Eeg-Based Upper Limb Exoskeleton.

Method Reference No; Year	Targeted Regions	No. of DOF	Types of Sensors Used	Advantages
Ref. [68] 2020	Hand	1- DOF	32 EEG channels (actiCAP)	Provide exoskeleton feedback
Ref. [49] 2022	Hand	1- DOF	14 electrodes Emotiv EPOC	Soft, adjustable, and customized
Ref. [72] 2020	Elbow, forearm, and wrist	5- DOF	12 EEG electrodes	High torque and rigid
Ref. [74] 2020	Wrist	1- DOF	7 EEG channels (EASYCAP)	Low-cost and lightweight.
Ref. [69] 2021	Hand	2- DOF	16 EEG channels (actiCAP)	Low-cost and lightweight, portable, Ergonomic and simple.
Ref. [70] 2021	Hand	1- DOF	11 active electrodes (g.LADYbird).	Exactly fitted to the patients' hands.
Ref. [71] 2022	Hand	1- DOF	16 EEG channels (Shenzhen Rehab Medical Technology)	Small size and wearable
Ref. [73] 2020	Hand and fingers	5- DOF	32-channels Quick-Cap	Provides passive, supportive and active modes, High flexibility, and enhances the movement and the strength of fingers

## 6 Hybrid control system for upper limb exoskeleton

EEG and EMG signals in combination will be included in this section as well as different measurement techniques and different exoskeletons for different regions like hand [75,76], arm [77,78], fingers [79], and wrist and hand [80], will be explained.

The Correlation between Band-limited Power Time-courses (CBPT) concerned with EEG and EMG was utilized in a novel corticomuscular feature extraction method. 8 hemiplegic patients and 16 healthy individuals were recruited for a BCI-based hand orthosis triggering function. The good subjects were evenly split into two groups; the experimental group for the BCI test based on the CBPT, and the control group for the BCI test based on the EEG-EMG coherence. The CBPT-based BCI system's group categorization accuracy for the patients was 84:53 ± 4:58% and for the healthy experimental group was 92:81 ± 2:09%. The EEG-EMG CBPT is a better substitute for a corticomuscular feature while operating a BCI system, according to the results [75]. A non-invasive BCI headset called "OpenBCI” with an open-source cost effective robotic arm (U-Arm) makes up a portable and inexpensive assistive technology controlled by BCI that is used to perform tasks associated with rehabilitation, like home use or adaptability. EEG and EMG are used in this system to control the arm. The findings demonstrated that EMG is an extremely reliable technique for assistive control technology [77]. Multimodal Interfaces (MMIs) system which included EEG, EMG, and electrooculography (EOG) to operate the delicate robotic hand was presented. 6 healthy individuals performed right and left-hand motor imaginary, both gazing left, and right eye movements, and various hand tasks in various procedures to control a soft robot in different movements. The MMIs can accomplish an average classification accuracy of 93.83%. The findings demonstrate that the number of MMI control instructions is higher than what can be achieved with any one modality [76]. EMG analysis and Corticomuscular Coherence (CMCoh) were selected to consider the corticomuscular arrangement pattern of the proximal upper extremity (UE) compensation measures when performing distal UE tasks in a chronic victim. 10 age-matched unimpaired controls and 14 chronic stroke patients performed isometric finger flexions and extensions when the strongest voluntary contractions are at 20 and 40%. EEG and EMG signals were captured from the sensorimotor region and associated finger muscles respectively to explore the Beta band's CMCoh peak values. Considerable variations (*P* < 0.05) were noticed in both maximum Flexor Digitorum (FD) and Extensor Digitorum (ED) CMCoh involving the two groups during finger extensions. The stroke patients exhibited substantial changes in peak Triceps Brachii (TRI) and Biceps Brachii (BIC) CMCoh, while the unimpaired controls showed intragroup variability between 20 and 40% levels for peak FD and ED CMCoh (P 0.05). TRI and BIC muscles were shown greater EMG activation levels in EMG parameters (*P* < 0.05), and higher Contraction Index (CI) amounts in the muscle groups requiring TRI and BIC as all the flexion and extension movements compared to those in the control group in the stroke group (*P* < 0.05) [79]. In time with the EMG data of the unilateral arm, a robotic arm control system gathered the EEG signals of the right and left-hand motor imagery. Each motion was recognized with an average accuracy of more than 94%. By using an EEG electrode, four channels' EEG signals C3, C4, FZ, and A1 were captured. A total of 10 channel EMG electrodes were employed by adopting the ring attachment method. Gesture tasks involve wrist extroversion, wrist introversion, fist clenching, finger kneading, rest, and hand opening. Each movement in the EMG training lasts 2 s in single trials, for a total of 12 s over six motions [78]. In order to unite the central and peripheral Voluntary Motor Efforts (VMEs) in neuromuscular systems in stroke patients, CMCoh and EMG-driven control were proposed. The NMES robot system enhanced the CMC-EMG-driven control to direct and assist stroke patients with wrist-hand flexion and extension. A trial of 20 treatment sessions for a pilot single-group was performed with the enhanced system to evaluate the probability of the wrist-hand procedure on the 16 chronic strokes. Accurate wrist-hand rehabilitation was accomplished by the developed system, submitting restrained cerebral and muscle compensation from the proximal UE and the contralesional hemisphere, and improved central-and-peripheral VME allocation on UE muscles [80]. Researchers looked into how oscillatory sensorimotor brain activity relates to motor recovery. The neurophysiological data collected from 30 stroke patients with severe upper-limb paralysis served as the foundation for this investigation. Using multilayer linear predictive modeling, the alpha oscillations over the motor cortex of 22 patients were investigated. Between clinical improvement and the development of the alpha desynchronization when therapeutic intervention was taking place, a significant correlation was identified. Patients who began the program with a significant alpha desynchronization had their alpha desynchronization raised. Whereas, Patients improved if they reduced alpha desynchronization more on both hemispheres at the beginning of the training stages. The findings demonstrate the interhemispheric stability of initial alpha desynchronization and its importance in motor recovery for the stratification of stroke patients undergoing BMI treatments [81]. A brief comparison is in Table IV.

**Table IV:** A Brief Description of Emg + Eeg-Based Upper Limb Exoskeleton.

Method Reference No; Year	Targeted Regions	No. of DOF	Types of Sensors Used	Advantages
Ref. [75] 2018	Hand	4- DOF	6 EEG electrodes+2- sEMG	Lightweight, easy to wear, and portable.
Ref. [77] 2019	Arm	3- DOF	6 EEG electrodes of (OpenBCI headset)+ 2 electrodes of (OpenBCI headset) for EMG	Open source and adaptable
Ref. [76] 2019	Hand	9- DOF	6 EEG electrodes (Neuroscan nuamps Express system)+Myo Armband	Wearable, flexible and comfortable
Ref. [79] 2020	Fingers	1- DOF	21 channels (64-channel g.GAMMAsys)+ Four pairs of sEMG	Wearable and lightweight
Ref. [78] 2021	Arm	6- DOF	4 EEG channels + 10 sEMG channels.	Compact and stable
Ref. [80] 2022	Wrist and hand	2- DOF	15 channels (64-channel g.GAMMAsys)+2-channel sEMG electrodes	Wearable and lightweight
Ref. [81] 2019	Arm	1- DOF	16 EEG channels (actiCAP)+ 8-channel sEMG electrodes	Provides five modes of operation and enhances patient motivation

## 7 EMG/EEG based control system for upper limb rehabilitation

In this section, EMG/EEG-based control systems involving electrical stimulation (ES) will be introduced.

A study to determine whether giving ES to chronic stroke patients before each hand task training session over the course of eight weeks can enhance their control over their neuromuscular system and hand function.12 subjects were recruited and randomly sampled into control and ES groups. The ES group underwent a 40-min ES on the median nerve of the paralyzed side before each training session. The two groups each got 20 min of hand task training two times a week. A rehabilitation program concerned with the eighth week performed ES sessions peripherally before function training can enhance hand function and neuromuscular control in stroke patients [82]. The Artificial Muscle Intelligence with Deep Learning (AMIDL) system, which integrates the patient's intentions with artificial muscle motions in an efficient manner to boost performance, was presented to help and rehabilitate paralyzed people. EEG sensors were used to capture human thoughts which were converted into body movements, by utilizing a microcontroller and Transcutaneous Electrical Nerve Stimulation (TENS) system. The recorded artificial muscle movements will stimulate the damaged body part if the acquired EEG signal drops below the necessary level. WHT transform was used for EEG signal feature extraction and classification [83]. A new artificial neural rehabilitation system was proposed, which combines Functional Electrical Stimulation (FES) and BCI techniques, for recovery of motor function of the limb beyond stroke. 32 chronic stroke patients were enrolled in clinical trials. Victims were randomly split into two groups the NMES and the BCI-FES. A comparison between groups according to the variations in outcome measures during the intervention was done, and the tendencies of ERD amounts according to EEG for the BCI-FES group were analyzed. The FMA-UE and Kendall Manual Muscle Testing (Kendall MMT) grades for the BCI-FES group increased more than those for the other group, according to the results, demonstrating the system's superiority and usefulness in clinical settings. The change in quantities of the laterality coefficient (LC) based on ERD (LCm-ERD) had a very substantial positive connection with the variation in FMA-UE (r = 0.6093, P = 0.012), which provides a theoretical foundation for investigating novel objective evaluation techniques [84]. The influence of FES feedback was suggested with a combined Action Observation (AO) and MI (AOMI)-based BCI to enhance upper limb tasks and change patterns of brain activity in people with chronic stroke. A male 53 years old enrolled in this study. Due to his left hemiparesis, he was unable to extend his fingers or wrists. The rehabilitation program consists of 3 sessions every week for 4 weeks straight. The FMA-UE score was enhanced from 34 to 46, and the active range of motion (AROM) of wrist extension was raised from 0 to 20°. Laterality coefficient (LC) amounts in the beta band headed to be slightly negative even if LC readings in the alpha band appeared to be favorable following the intervention [85]. The variations in corticomuscular coupling and brain FC patterns in stroke individuals during rehabilitation were proposed. Stroke patients' EEG and EMG recordings were created synchronously at baseline (BL), two weeks after BL, and four weeks after BL. It is shown that all brain functional network connection was enhanced, and network characteristic amount was raised during the rehabilitation of stroke subjects. The average corticomuscular phase transfer entropy (PTE) initially decreased and then increased, with the frontal lobe showing the most evident rise [86]. A neurofeedback treatment technique for ongoing motor development was improved that integrates electrical stimulation and visual scenes. Twenty subjects' EEG and EMG data were gathered to examine the association between EEG-EMG coupling and their neurophysiological responses. Root mean square (RMS) analysis, Event-Related Desynchronization (ERD), transfer entropy (TE) patterns, and other methods were used to figure out the response of the cortical muscle. In comparison to the primary states, the RMS and the ERD enhanced following extensive motor training. but, there was no considerable change in BCI achievement. Directional TE amounts showed the mechanism of the cortical muscle. These outcomes explained that combining EMG and EEG patterns to establish and assess a BCI-based motor treatment technique is functional [87].

## 8 Mechanical design

Different techniques were used to design the upper extremity exoskeleton for stroke rehabilitation. In article [61] a modularized device was developed comprising two parts: 3Dprinted fingertip components for cable routing and an actuated aluminum forearm splint. The splint limits wrist movement to transmit the motor force to the fingers. Robotic soft hand actuated with Shape-Memory Alloys (SMA) wires were used which were controlled with a microcontroller in the article [62]. 1 DoF exoskeleton for elbow movement, comfortable, lightweight, and strong material used to carry the arm's weight of the user [48]. The designed system in the article [65] is composed of 2 wearable components: the wrist/hand (50 g) and the elbow (158 g). These parts were linked to the attached (80 g) pump box carried on the upper limb. Furthermore, a control box that weighs (358 g) contained a rechargeable 12-V battery, and system control circuits could be carried on the waist. This system helps the patient to achieve extending and retracting arm functions like elbow flexion, wrist flexion with the hand folded, wrist flexion with the hand open, and wrist extension are the motions involved. Article [63] presented a RobHand robotic platform and the exoskeleton, which is built on a direct-driven under-actuated serial 4-bar linkage system, supports the hand fingers in flexion and extension movements. Both articles [67,74] aimed to design a 3D-printed wrist rehabilitation exoskeleton. In the first article, PLA thermoplastic material was used which has a lightweight and low cost. The design is composed of two parts: The first one is an illustration of the flexion/extension portion, while the second one is an illustration of the adduction/abduction portion. Whereas in the second article, the material PLAMAX was used, and L16-P linear piston was used for actuation. The exoskeleton's purpose was to control of the position the wrist angle. Different parts were used in the article [46] such as the forearm, elbow joint, upper arm, servo motor, and high precision fitting parts. The PLA printing material was used, except for the planetary reducer and servo motor which form (1.5 kg) and (1 kg) respectively, the exoskeleton's overall structure weighs 0.53 kg. According to the article [45], the Wearable Robot (WR) has the most significant requirement like patient exoskeleton connection, exoskeleton volume, and motion range. The latter gives 3-DoF with a small volume and the servo motor actuator MX-106 was used. To achieve the exoskeleton design requirement, coupling with various users was suggested that depending on the forearm's standard deviation, the WR comes in two sizes: small 20.75 cm and medium 25.75 cm, as well as, the WR hand holder is available in two sizes, small 17.5 cm, and medium 19.5 cm, to accommodate a variety of hand sizes. The article [64] proposed an 8-DoF hand exoskeleton that was built utilizing 3D printing and has free finger movement. Both exoskeletons in the article [47,64] provide bilateral rehabilitation. In the article [68] exoskeleton Omega was used to develop the desired movement according to EEG signal. Elbow training was targeted in the article [72] including elbow flexion and extension movements by using the MAHI Exo-II exoskeleton. Hand Exoskeleton for Rehabilitation Objectives (HERO) in the article [69] is an exoskeleton made utilizing 3D-printed components with porous textiles integrated. In the article [70] the afflicted hand was fitted with the robotic hand orthosis of the stroke patient to provide passive movement feedback. RHB-III with 16 EEG channels (Shenzhen Rehab Medical Technology) in the article [71] which includes a manus robot feedback that helps the affected hand used to achieve actual grasping/opening functions. A soft robotic glove was used in the article [49] to give hand open-close and finger flexion-extension. The orthotic hand in the article [75] was developed in-house which was lightweight, easy to don, and portable, to provide flexion and extension of the patient's index finger, middle finger, and thumb. In article [77] U-Arm is used to perform tasks associated with rehabilitation, like access to resources or home use. In article [76] soft robotic hand was used which has many advantages such as they allow precise performance of exercises as they have a continuously deformable structure, permit plastic bending, and provide positive interactions between robot and limb. A robotic hand orthosis in the article [79] includes a module for the palm and wrist and five separate finger assemblies were utilized to settle the wrist direct at a 0° position. The thumb finger was fixed at an angle of 180° at the MCP joint and 165° at the PIP joint. The index, middle, ring, and little fingers of the patients were settled at a position of 135° at the Metacarpophalangeal (MCP) joint and 135° at the Proximal Interphalangeal (PIP) joints, which was at 50% open of the robotic hand. Later, after putting on the robotic hand, the patient's upper limb was connected to the palm orthosis on the slab. This robotic hand weighs 500 g. In article [80] for wrist and hand rehabilitation, an NMES robot training system was deployed. ReoGo rehabilitation robot was used in the article [81] for arm and hand movements.

## 9 EMG/EEG measurement and control technique

**Fig. 5:**
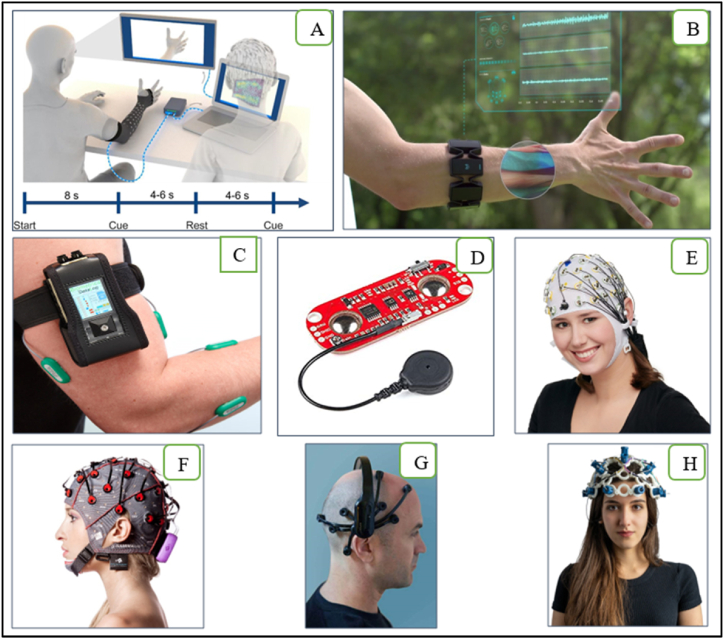
Examples of EMG/EEG electrodes: (A) NeuroLife EMG System [66], (B) Myo Armband [61], (C) datalog 4-channels [88], (D) MyoWare [89], (E) actiCAP 16-128-channels [90], (F) g.GAMMAsys [91], (G) Emotiv EPOC X 14-channels [92], (H) OpenBCI 16-channels [77].

Different detection methods and sensors were used to acquire EMG/EEG activities from stroke patients to control the associated exoskeleton for upper limb rehabilitation purposes. Examples of EEG/EMG electrodes are seen in Fig. 5 below.

EMG signals were recorded by using the surface EMG technique (sEMG) through MyoWare muscle sensors from Advancer Technologies in the article [48] and the pseudo-dynamic thresholding technique was used. In article [65], one sEMG is used to detect muscle activity from a patient's unaffected arm and if the signal value exceeds the threshold value, the FES will be delivered to the affected hand to promote desired motion. Myo Armband developed by Thalmic Labs was used in articles [46,61,62,64] to record EMG signals. 4 features were extracted: Standard Deviation (STD), Root Mean Square (RMS), Waveform Length (WL), and Mean Absolute Value (MAV). Maximum accuracy was obtained using RMS as a feature on the k-NN classifier. The EMG signal is recorded in the article [67] from the Extensor Carpi Ulnaris (ECU), by the MyoWare sensor to provide hand rehabilitation tasks. In article [66] 150 implanted electrodes in a wearable forearm sleeve and related software and hardware to record and decode sEMG from NeuroLife EMG System. In article [45] The sEMG activities were acquired utilizing a commercial Datalog sEMG equipment. These signals were captured from the biceps brachii long head, TRI long and lateral heads, Flexor Carpi Radialis (FCR), ECU, and Brachioradialis. In articles [68,81] EEG activity was recorded by utilizing 32-channel and 16-channel actiCap according to the10–20 international system configuration respectively. For the first article, EEG signals were recorded from regions TP10, CP6, CP2, CZ, C4, T8, FT10, FC6, FC2, F4, F8, FP1, FZ, F3, F7, FT9, FC5, FC1, T7, TP9, C3, CP5, CP1, PZ, P3, P7, O1, O2, P4, P8, TP10. Whereas, the second one, Fp1, Fp2, T7, T8, F3, Fz, F4, P3, Pz, P4, Cp3, Cp4, C3, Cz, C4, and Oz, and EMG signals were acquired by using 4 surface electrodes on each arm. In article [72] brain activity was recorded through EEG electrodes situated over the motor cortex, particularly, frontocentral (FCz, FC1 – FC4), central (Cz, C1– C4), and centro-parietal electrodes (CPz, CP1 – CP4). In article [74], 7 channels OpenBCI of continuous EEG were recorded positioned on C3, Cz, C4, F1, F2, P1, and P2 utilizing sintered ring electrodes placed in an EASYCAP EEG cap and the global 10–20 system. Also, AFz is used for the ground electrode and the right earlobe for the reference electrode. EMG signals were recorded using surface EMG electrodes. Brain activity in the article [69] was recorded with 16 active electrodes (actiCap) and was placed on FC3, CP3, CP4, C5, C3, C1, P3, Fz, FC4, Cz, C2, C4, P4, CPz, and Pz following the international 10-10 system. FCz was used for the reference electrode and AFz for the ground electrode. In article [70] 11 (g.LADYbird) active electrodes were used to record EEG signals and positioned on F3, Fz, F4, C3, C4, T3, P3, Pz, Cz, T4, and P4 following the international 10–20 system. The right earlobe used for the reference electrode and AFz for the ground electrode. In article [71] EEG was acquired using 16 unipolar Ag/Ag–Cl active electrodes within the 10–20 system positioned on F1, Fz, F3, FC3, FC1, FC2, FCz, FC4, CP1, CPz, CP2, C1, C2, C3, Cz, and C4. Emotiv EPOC was used in the article [49] to detect EEG signals. 32-channel (Quick-Cap) Ag/AgCl electrodes were used in the article [73] to acquire EEG signals following the 10–20 electrode placement system. The included electrodes for data acquisition are FP1, FP2, FC3, FC4, C3, CP3, Cz, C4, CP4, and CPz where FPz electrode is used as the ground electrode and the ipsilateral mastoid point was utilized as a reference electrode. In article [75] EEG channels from C3, Cz, and C4 were recorded in bipolar mode, based on 10–20 different international systems of the electrode. The EEG electrodes were positioned on FC3, CP3, FCz, CPz, FC4, CP4 regions, and the channels CP3, CP4, and CPz were bipolarly arranged with FC3, FC4, and FCz respectively. Bipolar EMG electrode placement was done on the right and left flexor digitorum superficialis. OpenBCI device in the article [77] with 8 EEG electrodes Pz, P3, P4, PO3, PO4, AF7, AF8, and Oz according to the 10–20 international system was used. Besides that, the InteraXon Muse BCI device with the EMG component was used for recording EMG signals on positions AF7 and AF8. In article [76] EOG and EEG activities were acquired utilizing a Neuroscan NuAmps Express system. To detect EEG activity and EOG movement, 40 Ag/AgCl electrodes were arranged in a flexible cap using the 10–20 international method. Myo Armband was used to track the movement of the arm depending on the EMG signal recorded from forearm muscles. A g.GAMMAsys 64-channel active electrodes of EEG cap in the article [79] was used according to the 10–20 system with electrodes positioned on C1, C2, C3, C4, C5, C6, FCZ, FC1, FC2, FC3, FC4, FC5, FC6, CPZ, CZ, CP1, CP2, CP3, CP4, CP5, and CP6 and grounded at AFz and referenced to the left earlobe. Four pairs of sEMG electrodes were used too to record signals from the TRI, ED, FD, and BIC. Four channels in the article [78] were used to collect EEG signals positioned on C3, C4, FZ, and A1 according to the 10–20 system. A1 and FZ were taken as the reference and ground electrodes respectively. 64-channel EEG capture cap. In the article [80], a GAMMAsys active electrode system was utilized to gather 15-channel EEG signals from electrodes positioned on Cz, CPz, FCz, C1-4, FC1-4, and CP1-4. Two-channel EMG signals were recorded from the joining of the ED and the ECU, and the muscle joining of the FD and FCR. In article [82] the 16-channel EEG (actiCap) was utilized to capture signals from positions FC1, FCz, FC2, FC3, FC4, C1, C2, C3, C4, CP3, CP1, CPz, Cz, CP2, CP4, and according to 10–20 system. EMG signals were recorded using sEMG. Human intentions in the article [83] were recorded in real-time by using 16-channel EEG sensors. TENS machine is combined with Muscle Inspired Algorithm (MIA) to create upper limb movements. EEG data in article [84] were recorded by using 32 electrodes positioned on F7, Fz, F4, F3, F8, FC3, FC1, FCz, FC4, FC2, P7, P3, Pz, P4, P8, TP7, TP8, C3, C1, Cz, C2, C4, CP3, CP1, CPz, CP2, CP4, O1, Oz, and O2 of the 10–20 system with F7 as reference electrode and P3 as ground electrode. In article [85], 16 active electrodes of EEG caps from g.Nautilus PRO were utilized. Electrodes were positioned on FP1, FP2, FC3, FC4, O1, O2, C5, C6, C3, C4, C1, C2, P3, P4, CP3 and CP4 with AFz for ground electrode and right earlobe for reference electrode.

Below is Table V which includes a comparison between different systems used.

**Table 5:** Comparison between different rehabilitation systems.

METHOD REFERENCE NO; YEAR	NO. OF SUBJECTS	TYPE OF SIGNAL	DEVICE ASSIGNED	TASK
**Ref.** [45] **2022**	2 Healthy subjects	EMG	Upper limb robotic exoskeleton	Elbow and wrist flexion/extension
**Ref.** [46] **2021**	20 Healthy subjects	EMG	upper arm robotic exoskeleton	Elbow flexion/extension
**Ref.** [68] **2020**	14 Stroke patients	EEG	Upper limb exoskeleton	Wrist extension
**Ref.** [47] **2019**	10 Healthy subjects	EMG	Elbow exoskeleton	Elbow flexion/extension
**Ref.** [48] **2019**	1 Healthy subject	EMG	Upper limb exoskeleton	Elbow flexion/extension
**Ref.** [49] **2022**	30 Stroke patients	EEG	Soft robotic glove	Fingers flexion/extension hand open/close
**Ref.** [61] **2018**	4 Stroke patients	EMG, pressure, and bend sensors	Robotic hand orthosis	Hand open/close
**Ref.** [62] **2017**	1 Stroke patient	EMG	Hand exoskeleton	Grasping/releasing an object
**Ref.** [65] **2020**	15 Stroke patients	EMG	Upper limb exoskeleton	Reaching/withdrawing
**Ref.** [63] **2023**	18 Healthy subjects	EMG	Robotic hand	Hand gestures
**Ref.** [67] **2020**	10 Stroke patients	EMG	Wrist exoskeleton	Hand flexion/extension/adduction/abduction
**Ref.** [66] **2022**	7 Stroke patients	EMG	Wearable forearm sleeve	Hand/wrist/forearm movements
**Ref.** [64] **2022**	7 Stroke patients	EMG	Hand exoskeleton	Grasping tasks
**Ref.** [72] **2020**	10 Stroke patients	EEG	Elbow, forearm, and wrist exoskeleton	Wrist flexion/extension, wrist radial/ulnar deviation, forearm pronation/supination, and elbow flexion/extension
**Ref.** [74] **2020**	11 Healthy subjects	EEG	Wrist exoskeleton	Wrist extension
**Ref.** [69] **2021**	1 Healthy subject	EEG	Hand exoskeleton	Fingers flexion/extension
**Ref.** [70] **2021**	10 stroke patients	EEG	Robotic hand	Fingers flexion/extension
**Ref.** [71] **2022**	18 Stroke patients	EEG	Robotic hand	Grasping/opening tasks
**Ref.** [73] **2020**	1 Stroke patient	EEG	AMADEO rehabilitation device	Hand movements
**Ref.** [75] **2018**	8 Hemiplegics; 16 healthy subjects	EMG + EEG	Hand orthosis	Grasping tasks
**Ref.** [77] **2019**	4 Healthy subjects	EMG + EEG	Robotic arm	Arm movements
**Ref.** [76] **2019**	6 Healthy subjects	EMG + EEG	Soft robotic hand	Hand gestures
**Ref.** [79] **2020**	10 Healthy subjects; 14 stroke patients	EMG + EEG	Robotic hand	Fingers flexion/extension
**Ref.** [78] **2021**	10 Healthy subjects	EMG + EEG	Robotic arm	Hand gestures
**Ref.** [80] **2022**	16 Stroke patients	EMG + EEG	NMES Robotic hand	Hand flexion/extension
**Ref.** [81] **2019**	30 Stroke patients	EMG + EEG	ReoGo rehabilitation robot	Arm and hand movements
**Ref.** [82] **2018**	12 Stroke patients	EMG + EEG	Peripheral electrical stimulation (ES**)**	Arm and hand movements
**Ref.** [83] **2019**	10 Healthy subjects; 10 stroke patients	EMG + EEG	TENS device	Hand gestures
**Ref.** [84] **2021**	32 Stroke patients	EEG	BCI-FES system	Wrist extension
**Ref.** [85] **2017**	32 Healthy subjects	EEG	BCI-FES system	Wrist extension
**Ref.** [86] **2022**	3 Stroke patients	EMG + EEG	Brain functional network and corticomuscular coupling	Upper limb rehabilitation
**Ref.** [87] **2022**	20 Healthy subjects	EMG + EEG	BCI-FES system	Upper limb rehabilitation

## 10 Conclusion

Despite the study of robotic exoskeletons beginning in the 1960s, the continuous modification and development of these systems are still persistent. After reviewing the literature mentioned above, we concluded that:

• One of the most significant biological signals that directly reflects the actions of human muscles is the EMG signal. As the EMG signals from the subject's muscles directly represent the subject's motion intentions, EMG-based control is one of the most successful control strategies for multiple kinds of rehabilitative robotic systems.
• Multi-DOF power-assist exoskeletons are difficult to implement with EMG-based control.
• EMG signals acquired from paralyzed limbs are mostly weak, which negatively affects motion intention detection.
• EEG signal has been excessively serving as a tool for treatment and estimation of rehabilitation strategies because of its noninvasive and easy measurement of human brain activity.
• EEG provides a method for analyzing the synchronization between muscle activity and motor cortex during movement.
• The EEG-based neurofeedback method is broadly utilized as a tool for improving the cognitive, motor, and psychological functions of individuals.
• A robotic exoskeleton's creation and implementation that is governed by EMG and EEG signals is performed as closely as possible to the human movement in different functions.
• By depending on EEG and EMG signals occurring simultaneously, accurate results and a shorter rehabilitation time are achieved.
• Soft exoskeletons are more comfortable wearing than hard ones but have less precise control movement.
• Increasing the size of the robotic exoskeleton mostly adds more burden to the patient's limb.
• Motor recovery after stroke depends on active engagement and involvement of the patient during rehabilitation to improve activity-dependent neuroplasticity. EEG/EMG-based robotic exoskeleton has performed better patient engagement and achieved better results than neuromuscular stimulation alone or, robot-assisted. Increasing the sample of patients, the frequency of interventions, and the duration of treatment time is important to accurately measure the variations in recovery.

Many technical developments that can improve the clinical capabilities of EEG/EMG-based controlled robotics for upper limb stroke rehabilitation have been cited. Additional studies and large-scale clinical assessments are important to fully take advantage of EEG/EMG-based exoskeleton in motor control and rehabilitation.

## Author contribution statement

All authors listed have significantly contributed to the development and the writing of this article.

## Data availability statement

No data was used for the research described in the article.

## Additional information

No additional information is available for this paper.

## Declaration of competing interest

The authors declare that they have no known competing financial interests or personal relationships that could have appeared to influence the work reported in this paper.
